# ATLAS: protein flexibility description from atomistic molecular dynamics simulations

**DOI:** 10.1093/nar/gkad1084

**Published:** 2023-11-20

**Authors:** Yann Vander Meersche, Gabriel Cretin, Aria Gheeraert, Jean-Christophe Gelly, Tatiana Galochkina

**Affiliations:** Université Paris Cité and Université des Antilles and Université de la Réunion, INSERM, BIGR, F-75014 Paris, France; Université Paris Cité and Université des Antilles and Université de la Réunion, INSERM, BIGR, F-75014 Paris, France; Université Paris Cité and Université des Antilles and Université de la Réunion, INSERM, BIGR, F-75014 Paris, France; Université Paris Cité and Université des Antilles and Université de la Réunion, INSERM, BIGR, F-75014 Paris, France; Université Paris Cité and Université des Antilles and Université de la Réunion, INSERM, BIGR, F-75014 Paris, France

## Abstract

Dynamical behaviour is one of the most crucial protein characteristics. Despite the advances in the field of protein structure resolution and prediction, analysis and prediction of protein dynamic properties remains a major challenge, mostly due to the low accessibility of data and its diversity and heterogeneity. To address this issue, we present ATLAS, a database of standardised all-atom molecular dynamics simulations, accompanied by their analysis in the form of interactive diagrams and trajectory visualisation. ATLAS offers a large-scale view and valuable insights on protein dynamics for a large and representative set of proteins, by combining data obtained through molecular dynamics simulations with information extracted from experimental structures. Users can easily analyse dynamic properties of functional protein regions, such as domain limits (hinge positions) and residues involved in interaction with other biological molecules. Additionally, the database enables exploration of proteins with uncommon dynamic properties conditioned by their environment such as chameleon subsequences and Dual Personality Fragments. The ATLAS database is freely available at https://www.dsimb.inserm.fr/ATLAS.

## Graphical Abstract

**Graphical Abstract:**
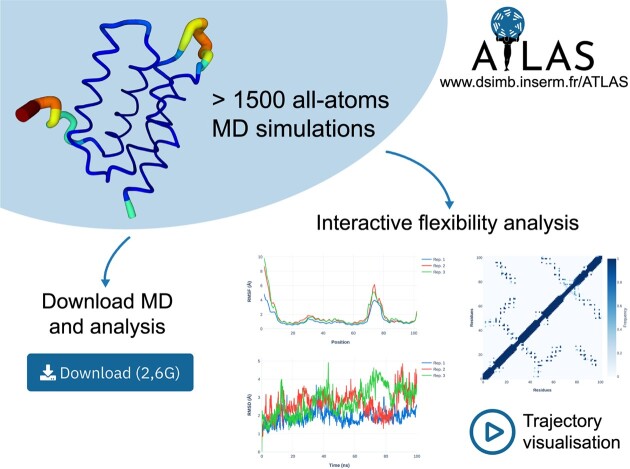

## Introduction

Proteins are dynamic entities that undergo continuous conformational changes of varying magnitudes, which are essential in biological processes such as enzyme catalysis, protein-protein interactions, and allosteric enzyme activation (1–3).

Information on protein flexibility can be obtained using experimental methods, such as X-ray crystallography (B-factor) or NMR spectroscopy (order parameter), However, these methods have limitations. First, experimental methods can only provide indirect information on protein dynamics without atomistic details on the corresponding transitions. Secondly, experimental conditions vary significantly, complicating comparison across experiments and are often far from the conditions expected *in vivo* (4–7). While the current revolution in the field of structural bioinformatics brought by the AlphaFold2 (8) release has significantly democratised access to static three-dimensional structures of numerous proteins, analysis and prediction of protein dynamics still remains one of the most important challenges, primarily due to the lack of reliable data (9,10).

During the last decades, molecular dynamics (MD) simulations were demonstrated to provide valuable information on protein conformational behaviour on both local and global scales (11–16). In particular, protein structure ensembles generated using MD trajectories of tens of nanoseconds enhance docking performance (17–21), allow detection of pockets participating in protein-protein interaction (22) or detect flexibility patterns characteristic for residues involved in protein-protein interface formation (23). MD simulations lasting for hundreds of nanoseconds allow detection of allosteric pathways (24–26), while longer MD can bring valuable insights on major conformational changes (27,28). Nevertheless, systematic comparative analysis of simulations conducted by different research groups is significantly complicated by the divergence in the system settings, MD simulations protocols as well as software and force field used for the simulations. To overcome this issue, several initiatives have led to the release of public databases for specific protein classes. Examples include MemProtMD (29), which focuses on coarse-grained simulations of membrane proteins, GPCRmd (30), which gathers trajectories of G protein-coupled receptor (GPCR) proteins, and SCoV2-MD (31), gathering simulations and analysis of SARS-CoV-2 proteins. Prior to our work, only two databases attempted to provide general datasets of MD for soluble proteins: MoDEL (32) and Dynameomics (33). However, only MoDEL is still accessible, though it is only partially functional and no longer updated. Dynameomics contained native state as well as unfolding dynamics, but is currently inaccessible. Additionally, both databases face issues, the major one being the lack of a uniform protocol, necessary for rigorous comparison between multiple protein simulations, and replicates for ensuring model reliability.

Here, we present ATLAS, a database of standardised all-atom molecular dynamics simulations on a large set of representative protein structures. All the trajectories, their analyses, as well as their biological annotations are freely accessible online in the form of a website containing interactive diagrams and trajectory visualisation at https://www.dsimb.inserm.fr/ATLAS. ATLAS consists of three datasets of molecular dynamics simulations. Currently, the main ATLAS dataset comprises 1390 protein chains, carefully chosen to provide an exhaustive sampling of the conformational space within the Protein Data Bank (PDB) (34). Two other datasets focus on proteins with specific dynamics behaviour. First one reports MD of 100 proteins containing Dual Personality Fragments (DPFs). DPFs are protein regions that can exist in both disordered and ordered states within different crystallographic structures of the same protein (35). The transition of a DPF to an ordered state is often associated with the presence of a protein partner or interaction with a ligand. The second one reports dynamics for 32 proteins containing chameleon sequences (36), which can adopt a different ordered secondary structure conformation (α-helix or β-strand) in different proteins. Both chameleon sequences and Dual Personality Fragments are of great biological interest and the understanding of their dynamic properties can bring new information on the mechanism of the corresponding protein function and evolution.

## Materials and methods

### Protein selection

#### Representative dataset (ATLAS dataset)

High-quality protein chains from the PDB (version of July 2022) were thoroughly filtered to ensure structural diversity by removing redundancy in terms of X-class ECOD (37) domains (indicating similar fold and possible homology). We have first selected all X-ray structures of protein chains of at least 38 residues long with resolution below or equal to 2 Å, in accordance with MolProbity's quality thresholds (38). We have filtered out proteins without an ECOD ID (v285) and membrane proteins (consensus of OPM (39), PDBTM (40), MemProtMD (29), and mpstruc from RCSB PDB - April 2023) (41). We then selected the best chain for each ECOD X-class domain. To do so, proteins with more than 10 consecutive missing residues were excluded. Among the remaining proteins, we prioritised those present in the quality-filtered rotamer datasets Top8000 and Top2018 (38,42). We also gave priority to proteins crystalised in monomeric state by selecting first structures crystalised as monomers and predicted by PISA (43) as such, then monomeric structures predicted as multimeric by PISA and finally multimeric structures. In cases of multiple resolved structures, we selected one of them based on i) the lowest number of consecutive gaps, ii) the biggest sequence length, and iii) the lowest proportion of gapped positions with respect to the protein sequence length. Therefore, the ATLAS dataset contains 1068 proteins with 1149 strictly non-redundant X-class ECOD domains, which we will further refer to as ‘non-redundant core’.

For 322 proteins of the non-redundant core, we have performed MD simulations of the alternative high-quality protein structures sharing the same ECOD X-class. The main goal of ATLAS is to provide several representative dynamics per structural class and progressively expand it. For today, the ATLAS dataset contains MD trajectories for 1390 different proteins.

#### Chameleon sequences

For all chameleon sequences of the ChSeq database (36) longer than 7 amino acids we have manually chosen those containing high-quality structures. We have selected two protein structures per chameleon sequence: one for helical and one for β-strand conformation.

#### Dual personality fragments

To identify proteins containing DPFs, we used the following protocol. First, we gathered all high-quality protein structures from the PDB with a resolution of 2 Å or better, matching the same protein sequence. Within each group of structures, we identified the largest continuous protein fragment that was observed in both the folded and disordered states (missing residues in the PDB file), excluding extremities. We then selected the best quality representative structure in the folded state. To ensure diversity, we filtered out proteins with sequences sharing more than 20% identity using MMseqs2 (44). From the remaining candidates, we selected 100 DPFs with lengths ranging from 8 to 20 amino acids, while sampling fragments that adopt alpha, beta and coil secondary structures in equal proportion. We used Dictionary of Secondary Structure of Proteins (DSSP) assignments to define these classes (45). A fragment with four consecutive helix residues or more is assigned to the alpha helix class, and one with 3 consecutive strand residues is allocated to the beta sheet class. If none of these conditions are verified, the fragment is assigned as coil.

### Protein structure preparation for MD simulations

All water and ligand molecules were removed from crystal structures to ensure protocol uniformity. Missing residues were modelled using MODELLER v10.1 (46) for proteins with no more than five consecutive gaps (or modified residues) and AlphaFold v2.1.0 (8) for proteins with 6–10 consecutive gaps in their resolved structures. These thresholds were chosen to maximise model reliability within reasonable computation time. Indeed, only 2.5% of the reconstructed residues with MODELLER have a low accuracy for five residue long loop reconstruction, and 0% below this threshold (47). For DPF and chameleon sequences, we used only MODELLER to complete missing residues.

### Molecular dynamics simulation protocol

All-atom molecular dynamics simulations were performed with GROMACS v2019.4 (48) with the CHARMM36m force field (July 2020 version), which was developed to provide a balanced sampling of folded and unfolded conformations for both folded and intrinsically disordered proteins (49) and provides extensive parameters for various compounds such as proteins, lipids and sugars (50). Each protein was placed in a periodic triclinic box, solvated using TIP3P water molecules, and neutralised with Na^+^/Cl^−^ ions at a concentration of 150 mM.

To optimise the system's geometry before the simulation, we performed energy minimisation using the steepest descent algorithm for 5000 steps. Subsequently, we conducted equilibration in a canonical ensemble (NVT) for 200 ps with a 1 fs time step. This was followed by equilibration in an isothermal-isobaric thermodynamic ensemble (NPT) for 1 ns with a 2 fs time step, employing the leap-frog integrator. The temperature was maintained at 300 K using the Nosé-Hoover thermostat with corrections applied every 1 ps (τT) for both the NVT and NPT ensembles. During NPT equilibration, we maintained the pressure at 1 bar using the isotropic Parrinello-Rahman barostat with a τ_p_ value of 5 ps. Throughout the minimisation and equilibration stages, heavy atom positions were restrained using a harmonic potential with force constant of 1000 kJ/mol/nm^2^. For all proteins density stabilisation was observed by the end of the first 100 ps of NPT equilibration with average values of 1045 kJ/mol/nm^2^. Subsequently, heavy atom restraints were released for the NPT production step, employing the same thermo- and barostat as for the NPT equilibration. The final production molecular dynamics simulations were carried out in three replicates using a different seed for the random starting velocities assigned from a Boltzman distribution. Each 100 ns replicate ran with a time step of 2 fs and atomic coordinates were saved every 10 ps. Covalent bonds involving hydrogen atoms were constrained using the LINCS algorithm in all the simulations. Long-range electrostatic interactions were managed using the Particle-Mesh Ewald (PME) method.

These calculations were performed on the Juliot-Curie's Irene Rome supercomputer (TGCC/CEA), utilising dual-processor compute nodes running at 2.6 GHz with 64 cores per processor. The simulations generated 13.2 Terabytes of raw data (.xtc and .trr files) for the 1522 protein chains in the three datasets. In total, considering the three replicates, this amounts to 456.6 μs of simulation time, encompassing 4566 trajectories of 100 ns, and corresponding to over one million simulated amino acids.

### Protein dynamics report and description

The obtained MD trajectories were subjected to various analyses to assess the overall behaviour of the protein and the local flexibility of its backbone. These analyses are presented on interactive web pages with downloadable data, such as metrics calculated from MD data, information available in the crystal structure as well as annotations from other biological databases. The following parameters are reported:

Global protein behaviour:

- Root mean square deviation (RMSD) (in Å): The RMSD measures the deviation of the protein structure from its initial conformation. It is calculated on the backbone atoms using GROMACS.
- Gyration radius (in Å): The gyration radius indicates the compactness of the structure, computed with GROMACS along simulation.
- Contact map: The contact map shows the pairwise distances between the closest residue's heavy atoms, computed with MDTraj (51) using a 4.5 Å threshold to define contacts.

Local flexibility of protein backbone:

- Root mean square fluctuation (RMSF) (in Å): The RMSF represents the standard deviation of atomic positions in the trajectory. It is calculated on α-carbons using GROMACS.
- Phi and Psi angles (in °): The two main dihedral angles of peptide bonds in the protein, Phi and Psi, are calculated for each frame of the trajectory using MDTraj.
- Entropy-based index Neq: The Neq quantifies the average number of protein blocks (PBs) (52) at a given position in the sequence, reflecting the local deformability of the backbone during the dynamics. It ranges from 1 to 16, indicating the number of observed PBs during the dynamics (1: No PB variation, 16: Fully random PB distribution). The assignment of protein blocks is based on Phi / Psi angles and processed with PBxplore (53).
- Secondary structure assignment: The DSSP assigns secondary structure elements into eight categories for each frame of the trajectory, determined using MDTraj.
- Experimental B-factor extracted from initial PDB files for α-carbons (in Å²): The *B*-factor reflects the attenuation of X-ray scattering due to thermal motion, capturing atom vibrations and static structural disorder.

To provide a comprehensive assessment of flexibility, additional information is included:

- Co-crystalised interactions: Residues interacting with co-crystallised protein chains, ligands, ions or nucleotides. An interaction is defined by a distance between the α-carbon of the target residue and any heavy-atom of the co-crystallised partner inferior to 6 Å.
- Protein domains: ECOD/SCOPe/CATH (37,54,55) domain assignments extracted from the downloadable version of the respective databases, as well as domains assigned using the local version of SWORD2 (56,57).
- Minimum TM-score between first and last conformation among three replicates: A custom metric estimating the deviation of the protein structure at the end of the trajectories from the starting conformation, calculated with TM-align (58) (higher value indicates greater stability).
- Minimum TM-score between most divergent conformations: A custom metric evaluating the distance between the most divergent conformations among the replicates, calculated with TM-align (higher value indicates better reproducibility).
- AlphaFold2 predicted local distance difference test (pLDDT): AlphaFold2 pLDDT, which is a per-residue prediction confidence metric rather than a flexibility measurement. Computed locally with AlphaFold2 Collab v1.5.1 (59).
- Other general properties extracted from the PDB or UniProt such as organism or experimental resolution.

The calculations on the MD trajectory other than RMSD and gyration radius, were conducted after truncating the first 100 ps of the dynamics. This truncation was implemented to reduce the noise arising from the release of constraints at the beginning of the simulation.

## Results

### Database content

In its current version, the main ATLAS dataset contains 1390 protein chains, enabling us to capture a wide range of protein motions. Indeed, we provide MD trajectories for 1149 protein domains with unique ECOD X-class (denoting possible homology). This covers 97 out of the 100 most common ECOD domains and thus 91% of proteins with available ECOD ID.

Although it does not currently include all 2458 identified folds in ECOD, the database contains every X-class ECOD domain with available structure satisfying our stringent criteria. 1309 folds not featured in the database are either found only in the membrane proteins or do not have any representative X-ray structure of high resolution in the current version of the PDB.

**Figure 1.**
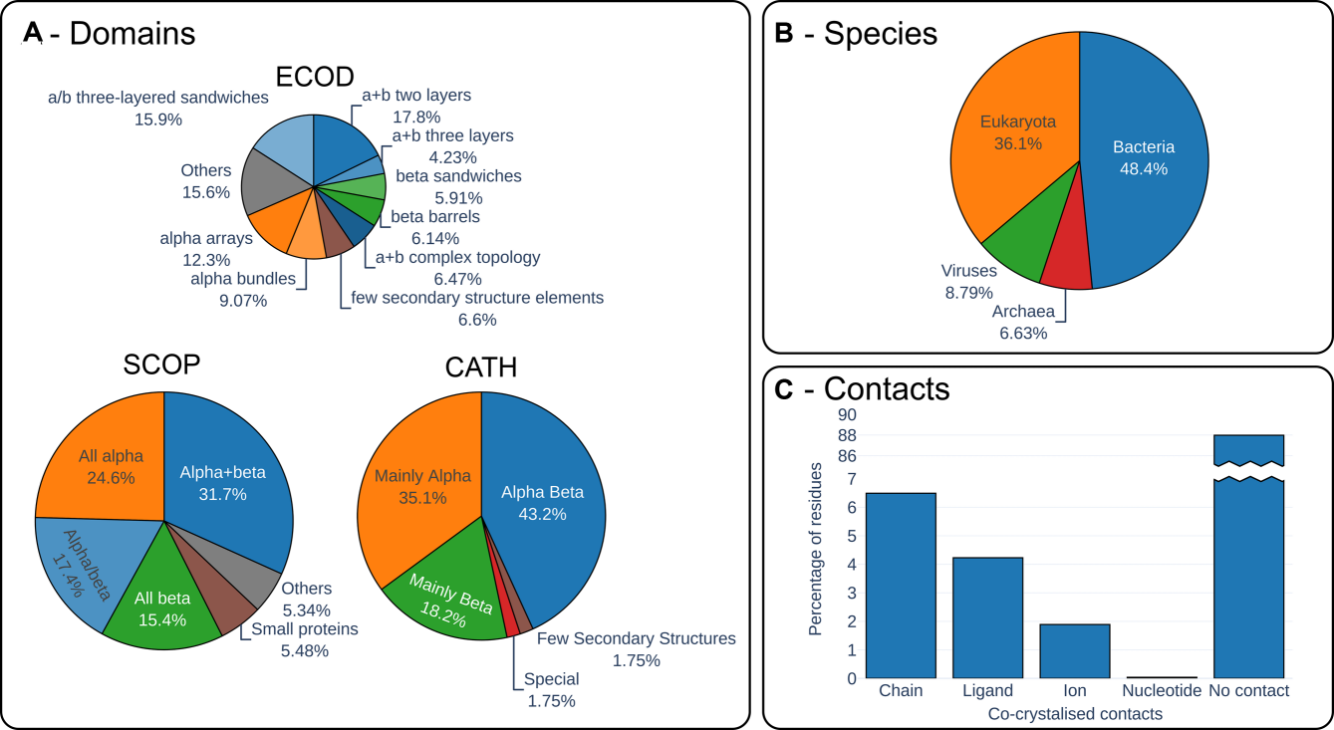
ATLAS main database content in terms of different protein domains (**A**), native species (**B**) and contacts found in crystal structures (**C**).

From the point of view of structural diversity, we cover a wide range of different folds from all-alpha to all-beta structures (Figure 1A) and protein sizes varying from 38 to 2128 residues (Figure S1A) and resolution from 0.72 to 2.0 Å (Figure S1B). The majority of proteins reported in ATLAS come from bacteria or eukaryotes (Figure 1B). Nevertheless, the database also contains proteins from archaea and viruses with original folds. Coiled regions correspond on average to 40% of protein residues per protein (Figure S1C). Finally, we report almost 6% of protein residues forming an interface with other chains in the crystal structure, while 4%, 2% and less than a percent of the residues were found in interaction with a ligand, ion or nucleotide respectively (Figure 1C). For DPF and chameleon protein regions this proportion increases significantly, therefore highlighting the role of intermolecular interaction in their stabilisation (Figure S2A, B).

Among simulated proteins, the majority demonstrates a rather modest deviation from the initial conformation with minimal TM-score between any frame of the trajectory and starting conformation of around 0.8 (Figure S1E). Minimal TM-score between starting and final conformation among replicates is higher than 0.9 for 32% of proteins indicating that conformational fluctuations during the simulation were reversible (Figure S1D) and the majority of protein structures tend to stabilise along the simulation (Figures S3 and S4).

### Browse by structural domains

Users have an option to explore the ATLAS dataset by domains using the ECOD, SCOPe or CATH domain classifications. The Browse page presents collapsible trees for easy navigation.

### Search in the database

Three methods can be used to search for a protein in the ATLAS database.

#### Search by features

**Figure 2.**
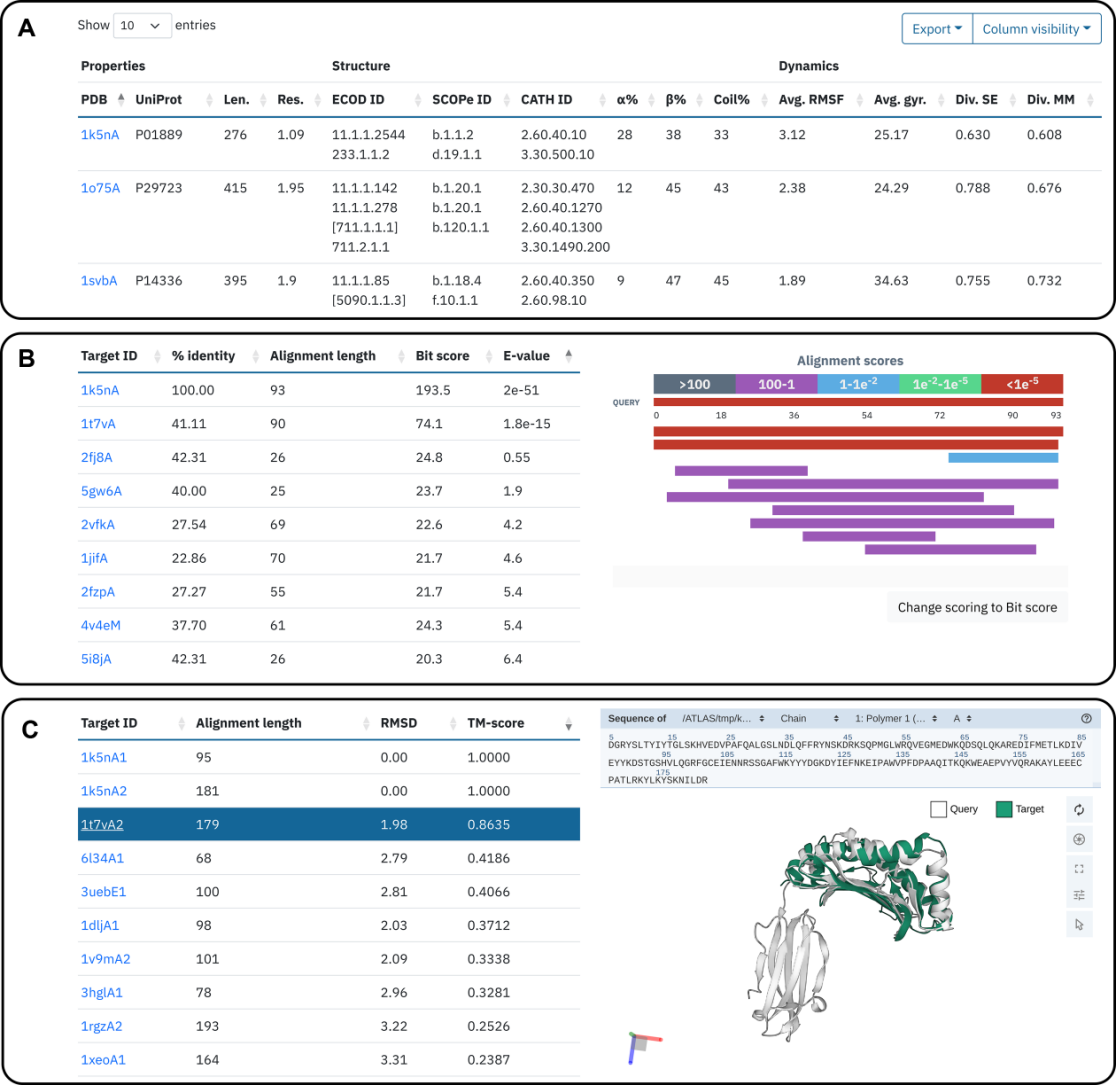
Example of search outputs. (**A**) Search by features, (**B**) search by sequence and (**C**) search by structure.

This procedure enables users to filter the database using protein dynamics descriptors (such as average RMSF, average Neq, conformational divergence during trajectories) as well as general protein properties gathered from external databases (e.g. domain classifications, UniProt/PDB annotations). The results are presented in a user-friendly table format and can be exported as a text file. An advanced search builder is also provided, allowing users to create more complex filtering rules (Figure 2A).

#### Search by sequence

The sequence search system allows users to query a protein sequence against the different databases to find similar proteins using local-global, local-local, and global-global search methods, from fasta36 v36.3.8 software (60). The alignment results table displays clickable target IDs, percentage of identity, alignment score, bit-score, and E-value of the matches with an *E*-value lower than 10. Additionally, a graphical summary of the alignment is provided for a quick overview of aligned regions and alignment quality (Figure 2B).

#### Search by structure

With the structure search approach, users can query a protein structure to find similar folds in ATLAS database using ProDy (61) to extract the requested protein chain and Kpax 5.1.3 (62) for structure alignment. They have the option to search for entire proteins in the three databases or by ECOD domains specifically present in the ATLAS dataset, useful for querying multi-domain proteins. ‘Flexible alignment’ option is available, allowing to flexibly superpose the target structure over the rigid query structure to account for backbone fluctuations. The alignment results table includes clickable target IDs, alignment length, RMSD, and TM-score of the top 10 best matches. Besides, a 3D structure viewer is provided to visualise the alignment between the query (displayed in white) and the target (displayed in green) (Figure 2C).

### Protein page

#### Page header

**Figure 3.**
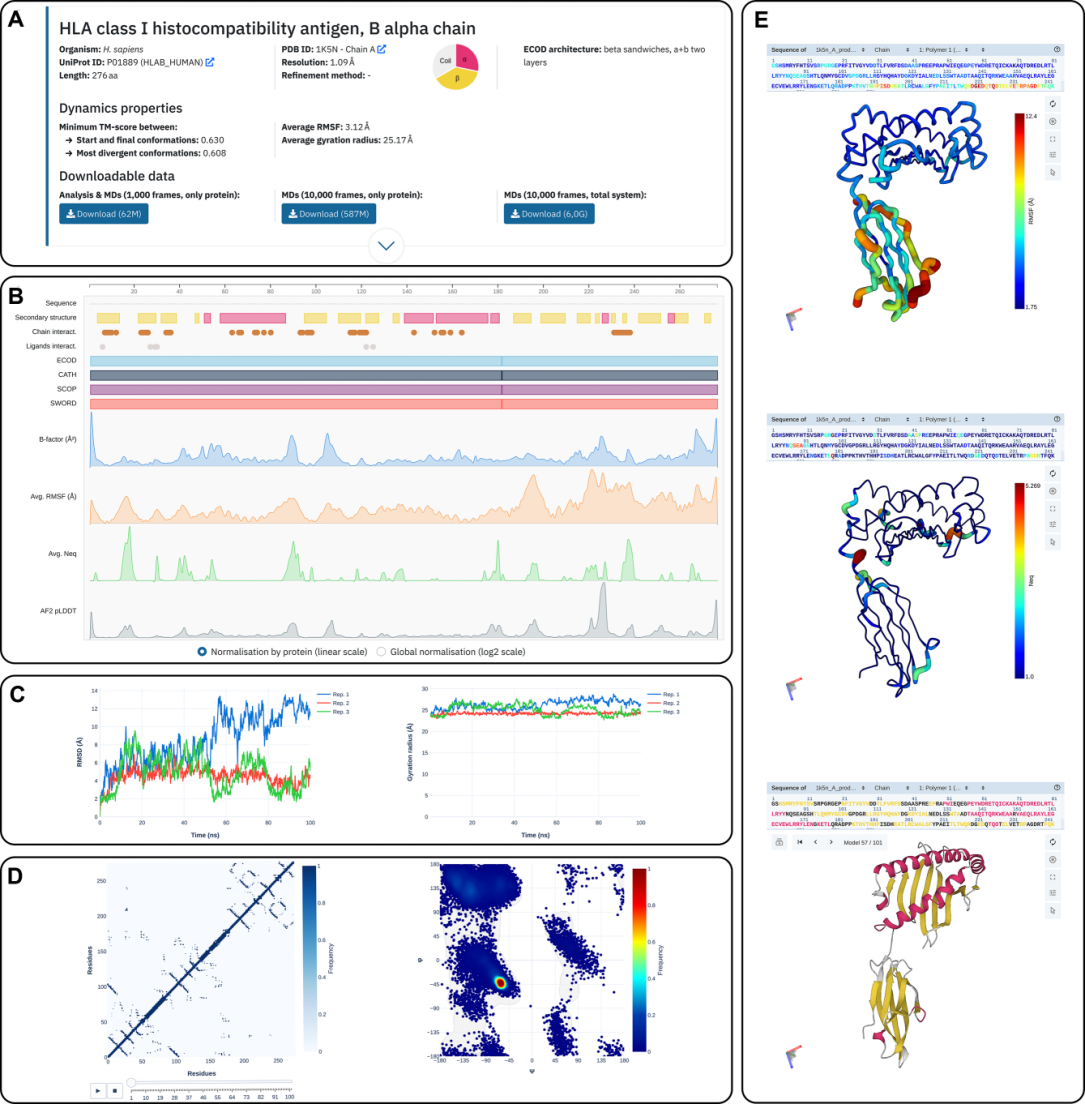
Example of protein page (PDB ID: 1k5n chain A). (**A**) Page header with annotations and downloadable data. (**B**) General residue-wise characteristics. (**C**) Replicates overview (left: RMSD, right: Gyration radius). (**D**) Detailed analysis (left: animated contact map, right: Ramachandran plot). (**E**) Examples of available structure visualisations in the Detailed analysis section (Replicate no. 1 – top: RMSF coloration, middle: Neq coloration, bottom: visualisation of the MD trajectory coloured by initial secondary structure).

This section displays information from external databases and programs, such as UniProt ID, secondary structure content, and domain delineations. It also provides general parameters computed from the molecular dynamics simulations, such as average RMSF and the minimum TM-score between the start and final conformations. Users can download trajectory data as a .zip archive using the ‘Download’ buttons in reduced format with the corresponding analyses (1000 frames for each replicate with solution molecules removed) and in complete format (10 000 frames for each replicate) either with or without solvent (Figure 3A).

#### General properties

Here, users can obtain an overview of main protein properties averaged over the replicates. The section includes visualisations of secondary structures, protein domain delineations (ECOD, CATH, SCOPe and SWORD2), experimental B-factor values, flexibility profiles (RMSF and Neq) averaged over the three MD replicates, and AlphaFold2 Collab pLDDT. Detailed values and positions in the sequence (the author-specified numbering extracted from PDB and a sequential numbering that starts from 1) are accessible on mouse hover. In addition, users can choose between min–max normalisation of flexibility profile from the protein only, for a better view of the subtle variations, or min-max normalisation of flexibility profile from the whole database (in log_2_ scale) to compare flexibility profiles of different proteins (Figure 3B).

#### Replicates overview

In this section, detailed flexibility profiles (RMSF, B-factor, Neq + pLDDT) and global analyses of protein conformational mobility during the simulation (RMSD and gyration radius) are provided. Users can view the diagrams for different replicates together or individually by clicking on the legend, and zoom in on specific regions of interest through click-and-drag functionality (Figure 3C).

#### Detailed analysis

To delve into the detected conformational changes, users can visualise the structure and MD trajectory, contact maps, Ramachandran plots and DSSP plots of each replicate. In the 3D viewers, protein can be coloured by sequence position or initial secondary structure. Flexibility visualisation is available either on the structure, modifying both the colour and width of the structure, or simply by visualising the trajectory itself on the ‘Dynamics’ tab (Figure 3E). Besides, the animated contact map illustrates the formation and destruction of contacts along the trajectory, and Ramachandran plots ensure coherent conformations of most residues (Figure 3D).

In Supplementary Information, we provide detailed examples of ATLAS protein page analysis (section ‘Examples of the protein page analysis’). We tackle the issue of inter-domain hinges and analysis of co-crystallised partners effects on the example of human HLA class 1 (Figures S5–S8) histocompatibility antigen as well as analyse dynamics of a Dual Personality Fragment located near the active site of hypoxanthine-guanine phosphoribosyltransferase (Figures S9–S12).

### Downloadable data

A Download page provides access to the GROMACS molecular dynamics protocols (.mdp) and force field files (CHARMM36m), the list of proteins composing different datasets, as well as parsable content of the protein page annotations for a more advanced protein selection. This page is also used to keep track of the database updates.

### REST API

Database data can also be accessed programmatically using the REST API documented in the ‘API’ tab. Users can download protein simulation data in the three possible formats (‘analysis’, ‘protein’ and ‘total’), as well as protein page summary in .json format available for each entry. API also allows the users to search for a protein in the datasets by sequence and by structure (see ‘Search in the database’ for the details), to download the MD parameters as well as to dump the latest release of the parsable version of the database (see ‘Downloadable data’ section).

## Discussion and perspectives

ATLAS database provides all-atom molecular dynamics simulations representative of the structural diversity of the PDB. Standardised protocol for protein selection and MD simulations followed by a thorough analysis of the resulting trajectories provides a source of valuable and comparable information on protein dynamics at different scales. Indeed, in their natural environment, proteins exhibit dynamic properties potentially linked to interaction with various molecules, and to their biological function under various conditions. Our database captures possible scenarios of this inherent diversity, providing information which is not directly available from X-ray structures. Combination of the reported dynamics information with protein annotations and contacts reported in the experimental data could help to deepen our understanding of the protein sequence-structure-function relationships.

The main goal of ATLAS is to provide information on the expected protein flexibility profile in solution and in absence of other molecular interactions. While three replicates of 100 ns simulations offer valuable insights into dynamics properties of proteins with a relatively stable structure, exploration of rare events or major conformational rearrangements of large proteins may require longer simulations. For now, the users can easily extend protein MD simulations using the last or the most divergent frames of the reported simulation as a starting point for further conformational sampling. In the medium-term perspective we will extend ATLAS database content both in terms of the simulation time and in terms of protein content. ATLAS will continue to expand to encompass emerging folds resolved in high-quality, as well as new representatives of existing folds with divergent sequences. In particular, the developed protocol for the selection of the representative high-quality protein structures will be regularly applied to the updated PDB content. Finally, the development of a unified MD repository such as the upcoming European initiative MDDB (https://mddbr.eu/) would be particularly beneficial for the expansion, sharing and the long-term sustainability of the ATLAS simulations.

The most interesting conclusions on protein dynamic-function relationships often depend on modelling intermolecular interactions. For now, accurate MD modelling of protein-ligand interactions requires extensive human expertise, incompatible with our automated and reproducible protocols, due to both lack of the adapted force field parameters covering chemical variability of different compounds and the problem of correct identification of the biologically relevant interactions (63). Nevertheless, our first specialised Dual Personality Fragment dataset has for purpose to shed light on conformational behaviour of the protein fragments particularly sensitive to ligand/partner removal. We will continue to expand ATLAS by adding several specialised datasets of MD simulations for proteins of particular biological interest, such as moonlight proteins. In the long-term perspective, such simulations will be completed by explicit MD simulations with protein partners as well as its post-translational modifications in order to explore their impact on protein dynamics, which is still poorly described for today. These expansions will enhance the diversity and scope of the ATLAS database, empowering users to explore a broader range of protein dynamics and behaviours.

## Supplementary Material

gkad1084_Supplemental_File

## Data Availability

The database website is freely available online without login requirement at https://www.dsimb.inserm.fr/ATLAS.

## References

[1] Kokkinidis M. , Glykos N.M., Fadouloglou V.E. Protein flexibility and enzymatic catalysis. Adv. Protein Chem. Struct. Biol. 2012; 87:181–218. 22607756 10.1016/B978-0-12-398312-1.00007-X

[2] Jubb H. , Blundell T.L., Ascher D.B. Flexibility and small pockets at protein-protein interfaces: new insights into druggability. Prog. Biophys. Mol. Biol. 2015; 119:2–9. 25662442 10.1016/j.pbiomolbio.2015.01.009 PMC4726663

[3] Teilum K. , Olsen J.G., Kragelund B.B. Functional aspects of protein flexibility. Cell. Mol. Life Sci. CMLS. 2009; 66:2231–2247. 19308324 10.1007/s00018-009-0014-6 PMC11115794

[4] Carugo O. How large B-factors can be in protein crystal structures. BMC Bioinf. 2018; 19:61. 10.1186/s12859-018-2083-8 PMC5824579 29471780

[5] Carugo O. Maximal B-factors in protein crystal structures. Z. Für Krist. - Cryst. Mater. 2019; 234:73–77.

[6] Carugo O. How anisotropic and isotropic atomic displacement parameters monitor protein covalent bonds rigidity: isotropic B-factors underestimate bond rigidity. Amino Acids. 2021; 53:779–782. 33928454 10.1007/s00726-021-02985-x PMC8128831

[7] Carugo O. B-factor accuracy in protein crystal structures. Acta Crystallogr. Sect. Struct. Biol. 2022; 78:69–74. 10.1107/S2059798321011736 PMC8725162 34981763

[8] Jumper J. , Evans R., Pritzel A., Green T., Figurnov M., Ronneberger O., Tunyasuvunakool K., Bates R., Žídek A., Potapenko A. et al. Highly accurate protein structure prediction with AlphaFold. Nature. 2021; 596:583–589. 34265844 10.1038/s41586-021-03819-2 PMC8371605

[9] Vander Meersche Y. , Cretin G., de Brevern A.G., Gelly J.-C., Galochkina T. MEDUSA: prediction of Protein Flexibility from Sequence. J. Mol. Biol. 2021; 433:166882. 33972018 10.1016/j.jmb.2021.166882

[10] Marchetti F. , Moroni E., Pandini A., Colombo G. Machine learning prediction of allosteric drug activity from molecular dynamics. J. Phys. Chem. Lett. 2021; 12:3724–3732. 33843228 10.1021/acs.jpclett.1c00045 PMC8154828

[11] Hansson T. , Oostenbrink C., van Gunsteren W. Molecular dynamics simulations. Curr. Opin. Struct. Biol. 2002; 12:190–196. 11959496 10.1016/s0959-440x(02)00308-1

[12] Collier T.A. , Piggot T.J., Allison J.R. Molecular dynamics simulation of proteins. Methods Mol. Biol. Clifton NJ. 2020; 2073:311–327. 10.1007/978-1-4939-9869-2_17 31612449

[13] Karplus M. , Petsko G.A. Molecular dynamics simulations in biology. Nature. 1990; 347:631–639. 2215695 10.1038/347631a0

[14] Lindorff-Larsen K. , Piana S., Dror R.O., Shaw D.E. How fast-folding proteins fold. Science. 2011; 334:517–520. 22034434 10.1126/science.1208351

[15] Gheeraert A. , Pacini L., Batista V.S., Vuillon L., Lesieur C., Rivalta I. Exploring allosteric pathways of a V-type enzyme with dynamical perturbation networks. J. Phys. Chem. B. 2019; 123:3452–3461. 30943726 10.1021/acs.jpcb.9b01294 PMC6604606

[16] Saltalamacchia A. , Casalino L., Borišek J., Batista V.S., Rivalta I., Magistrato A. Decrypting the information exchange pathways across the spliceosome machinery. J. Am. Chem. Soc. 2020; 142:8403–8411. 32275149 10.1021/jacs.0c02036 PMC7339022

[17] Santos L.H.S. , Ferreira R.S., Caffarena E.R. Integrating molecular docking and molecular dynamics simulations. Methods Mol. Biol. 2019; 2053:13–34. 31452096 10.1007/978-1-4939-9752-7_2

[18] Watanabe Y. , Fukuyoshi S., Kato K., Hiratsuka M., Yamaotsu N., Hirono S., Gouda H., Oda A. Investigation of substrate recognition for cytochrome P450 1A2 mediated by water molecules using docking and molecular dynamics simulations. J. Mol. Graph. Model. 2017; 74:326–336. 28475969 10.1016/j.jmgm.2017.04.006

[19] Terefe E.M. , Ghosh A. Molecular docking, validation, dynamics simulations, and pharmacokinetic prediction of phytochemicals isolated from Croton dichogamus against the HIV-1 reverse transcriptase. Bioinforma. Biol. Insights. 2022; 16:11779322221125604. 10.1177/11779322221125605 PMC9516429 36185760

[20] Tian S. , Sun H., Pan P., Li D., Zhen X., Li Y., Hou T. Assessing an ensemble docking-based virtual screening strategy for kinase targets by considering protein flexibility. J. Chem. Inf. Model. 2014; 54:2664–2679. 25233367 10.1021/ci500414b

[21] Wang B. , Buchman C.D., Li L., Hurley T.D., Meroueh S.O. Enrichment of chemical libraries docked to protein conformational ensembles and application to aldehyde dehydrogenase 2. J. Chem. Inf. Model. 2014; 54:2105–2116. 24856086 10.1021/ci5002026 PMC4114474

[22] Eyrisch S. , Helms V. Transient pockets on protein surfaces involved in protein-protein interaction. J. Med. Chem. 2007; 50:3457–3464. 17602601 10.1021/jm070095g

[23] Fornili A. , Pandini A., Lu H.-C., Fraternali F. Specialized dynamical properties of promiscuous residues revealed by simulated conformational ensembles. J. Chem. Theory Comput. 2013; 9:5127–5147. 24250278 10.1021/ct400486p PMC3827836

[24] Rivalta I. , Sultan M.M., Lee N.-S., Manley G.A., Loria J.P., Batista V.S. Allosteric pathways in imidazole glycerol phosphate synthase. Proc. Natl. Acad. Sci. U.S.A. 2012; 109:E1428–E1436. 22586084 10.1073/pnas.1120536109 PMC3365145

[25] Rivalta I. , Lisi G.P., Snoeberger N.-S., Manley G., Loria J.P., Batista V.S. Allosteric communication disrupted by a small molecule binding to the imidazole glycerol phosphate synthase protein–protein interface. Biochemistry. 2016; 55:6484–6494. 27797506 10.1021/acs.biochem.6b00859 PMC5283573

[26] Wurm J.P. , Sung S., Kneuttinger A.C., Hupfeld E., Sterner R., Wilmanns M., Sprangers R. Molecular basis for the allosteric activation mechanism of the heterodimeric imidazole glycerol phosphate synthase complex. Nat. Commun. 2021; 12:2748. 33980881 10.1038/s41467-021-22968-6 PMC8115485

[27] Klepeis J.L. , Lindorff-Larsen K., Dror R.O., Shaw D.E. Long-timescale molecular dynamics simulations of protein structure and function. Curr. Opin. Struct. Biol. 2009; 19:120–127. 19361980 10.1016/j.sbi.2009.03.004

[28] Ayaz P. , Lyczek A., Paung Y., Mingione V.R., Iacob R.E., de Waal P.W., Engen J.R., Seeliger M.A., Shan Y., Shaw D.E. Structural mechanism of a drug-binding process involving a large conformational change of the protein target. Nat. Commun. 2023; 14:1885. 37019905 10.1038/s41467-023-36956-5 PMC10076256

[29] Newport T.D. , Sansom M.S.P., Stansfeld P.J. The MemProtMD database: a resource for membrane-embedded protein structures and their lipid interactions. Nucleic. Acids. Res. 2019; 47:D390–D397. 30418645 10.1093/nar/gky1047 PMC6324062

[30] Rodríguez-Espigares I. , Torrens-Fontanals M., Tiemann J.K.S., Aranda-García D., Ramírez-Anguita J.M., Stepniewski T.M., Worp N., Varela-Rial A., Morales-Pastor A., Medel-Lacruz B. et al. GPCRmd uncovers the dynamics of the 3D-GPCRome. Nat. Methods. 2020; 17:777–787. 32661425 10.1038/s41592-020-0884-y

[31] Torrens-Fontanals M. , Peralta-García A., Talarico C., Guixà-González R., Giorgino T., Selent J. SCoV2-MD: a database for the dynamics of the SARS-CoV-2 proteome and variant impact predictions. Nucleic. Acids. Res. 2022; 50:D858–D866. 34761257 10.1093/nar/gkab977 PMC8689960

[32] Meyer T. , D’Abramo M., Hospital A., Rueda M., Ferrer-Costa C., Pérez A., Carrillo O., Camps J., Fenollosa C., Repchevsky D. et al. MoDEL (Molecular Dynamics Extended Library): a database of atomistic molecular dynamics trajectories. Struct. Lond. Engl. 2010; 18:1399–1409. 10.1016/j.str.2010.07.013 21070939

[33] van der Kamp M.W. , Schaeffer R.D., Jonsson A.L., Scouras A.D., Simms A.M., Toofanny R.D., Benson N.C., Anderson P.C., Merkley E.D., Rysavy S. et al. Dynameomics: a comprehensive database of protein dynamics. Struct. Lond. Engl. 2010; 18:423–435. 10.1016/j.str.2010.01.012 PMC2892689 20399180

[34] Berman H.M. , Westbrook J., Feng Z., Gilliland G., Bhat T.N., Weissig H., Shindyalov I.N., Bourne P.E. The Protein Data Bank. Nucleic Acids Res. 2000; 28:235–242. 10592235 10.1093/nar/28.1.235 PMC102472

[35] Zhang Y. , Stec B., Godzik A. Between order and disorder in protein structures: analysis of ‘dual personality’ fragments in proteins. Struct. Lond. Engl. 2007; 15:1141–1147. 10.1016/j.str.2007.07.012 PMC2084070 17850753

[36] Li W. , Kinch L.N., Karplus P.A., Grishin N.V. ChSeq: a database of chameleon sequences. Protein Sci. Publ. Protein Soc. 2015; 24:1075–1086. 10.1002/pro.2689 PMC4500308 25970262

[37] Schaeffer R.D. , Liao Y., Cheng H., Grishin N.V. ECOD: new developments in the evolutionary classification of domains. Nucleic Acids Res. 2017; 45:D296–D302. 27899594 10.1093/nar/gkw1137 PMC5210594

[38] Hintze B.J. , Lewis S.M., Richardson J.S., Richardson D.C. MolProbity's ultimate rotamer-library distributions for model validation. Proteins. 2016; 84:1177–1189. 27018641 10.1002/prot.25039 PMC4983197

[39] Lomize M.A. , Lomize A.L., Pogozheva I.D., Mosberg H.I. OPM: orientations of proteins in membranes database. Bioinforma. 2006; 22:623–625. 10.1093/bioinformatics/btk023 16397007

[40] Kozma D. , Simon I., Tusnády G.E. PDBTM: protein Data Bank of transmembrane proteins after 8 years. Nucleic Acids Res. 2013; 41:D524–D529. 23203988 10.1093/nar/gks1169 PMC3531219

[41] Bittrich S. , Rose Y., Segura J., Lowe R., Westbrook J.D., Duarte J.M., Burley S.K. RCSB Protein Data Bank: improved annotation, search and visualization of membrane protein structures archived in the PDB. Bioinforma. 2022; 38:1452–1454. 10.1093/bioinformatics/btab813 PMC8826025 34864908

[42] Williams C.J. , Richardson D.C., Richardson J.S. The importance of residue-level filtering and the Top2018 best-parts dataset of high-quality protein residues. Protein Sci. Publ. Protein Soc. 2022; 31:290–300. 10.1002/pro.4239 PMC8740842 34779043

[43] Krissinel E. , Henrick K. Inference of macromolecular assemblies from crystalline state. J. Mol. Biol. 2007; 372:774–797. 17681537 10.1016/j.jmb.2007.05.022

[44] Steinegger M. , Söding J. MMseqs2 enables sensitive protein sequence searching for the analysis of massive data sets. Nat. Biotechnol. 2017; 35:1026–1028. 29035372 10.1038/nbt.3988

[45] Touw W.G. , Baakman C., Black J., te Beek T.A.H., Krieger E., Joosten R.P., Vriend G. A series of PDB-related databanks for everyday needs. Nucleic. Acids. Res. 2015; 43:D364–D368. 25352545 10.1093/nar/gku1028 PMC4383885

[46] Webb B. , Sali A. Comparative Protein Structure Modeling Using MODELLER. Curr. Protoc. Bioinforma. 2016; 54:5.6.1–5.6.37. 10.1002/cpbi.3 PMC5031415 27322406

[47] Fiser A. , Do R.K., Sali A. Modeling of loops in protein structures. Protein Sci. Publ. Protein Soc. 2000; 9:1753–1773. 10.1110/ps.9.9.1753 PMC2144714 11045621

[48] Abraham M.J. , Murtola T., Schulz R., Páll S., Smith J.C., Hess B., Lindahl E. GROMACS: high performance molecular simulations through multi-level parallelism from laptops to supercomputers. SoftwareX. 2015; 1–2:19–25.

[49] Huang J. , Rauscher S., Nawrocki G., Ran T., Feig M., de Groot B.L., Grubmüller H., MacKerell A.D. CHARMM36m: an improved force field for folded and intrinsically disordered proteins. Nat. Methods. 2017; 14:71–73. 27819658 10.1038/nmeth.4067 PMC5199616

[50] Hollingsworth S.A. , Dror R.O. Molecular Dynamics Simulation for All. Neuron. 2018; 99:1129–1143. 30236283 10.1016/j.neuron.2018.08.011 PMC6209097

[51] McGibbon R.T. , Beauchamp K.A., Harrigan M.P., Klein C., Swails J.M., Hernández C.X., Schwantes C.R., Wang L.-P., Lane T.J., Pande V.S. MDTraj: a modern open library for the analysis of molecular dynamics trajectories. Biophys. J. 2015; 109:1528–1532. 26488642 10.1016/j.bpj.2015.08.015 PMC4623899

[52] de Brevern A.G. , Etchebest C., Hazout S. Bayesian probabilistic approach for predicting backbone structures in terms of protein blocks. Proteins. 2000; 41:271–287. 11025540 10.1002/1097-0134(20001115)41:3<271::aid-prot10>3.0.co;2-z

[53] Barnoud J. , Santuz H., Craveur P., Joseph A.P., Jallu V., de Brevern A.G., Poulain P. PBxplore: a tool to analyze local protein structure and deformability with Protein Blocks. PeerJ. 2017; 5:e4013. 29177113 10.7717/peerj.4013 PMC5700758

[54] Fox N.K. , Brenner S.E., Chandonia J.-M. SCOPe: structural classification of proteins–extended, integrating SCOP and ASTRAL data and classification of new structures. Nucleic Acids Res. 2014; 42:D304–D309. 24304899 10.1093/nar/gkt1240 PMC3965108

[55] Sillitoe I. , Bordin N., Dawson N., Waman V.P., Ashford P., Scholes H.M., Pang C.S.M., Woodridge L., Rauer C., Sen N. et al. CATH: increased structural coverage of functional space. Nucleic Acids Res. 2021; 49:D266–D273. 33237325 10.1093/nar/gkaa1079 PMC7778904

[56] Postic G. , Ghouzam Y., Chebrek R., Gelly J.-C. An ambiguity principle for assigning protein structural domains. Sci. Adv. 2017; 3:e1600552. 28097215 10.1126/sciadv.1600552 PMC5235333

[57] Cretin G. , Galochkina T., Vander Meersche Y., de Brevern A.G., Postic G., Gelly J.-C. SWORD2: hierarchical analysis of protein 3D structures. Nucleic Acids Res. 2022; 50:W732–W738. 35580056 10.1093/nar/gkac370 PMC9252838

[58] Zhang Y. , Skolnick J. TM-align: a protein structure alignment algorithm based on the TM-score. Nucleic Acids Res. 2005; 33:2302–2309. 15849316 10.1093/nar/gki524 PMC1084323

[59] Mirdita M. , Schütze K., Moriwaki Y., Heo L., Ovchinnikov S., Steinegger M. ColabFold: making protein folding accessible to all. Nat. Methods. 2022; 19:679–682. 35637307 10.1038/s41592-022-01488-1 PMC9184281

[60] Pearson W.R. , Lipman D.J. Improved tools for biological sequence comparison. Proc. Natl. Acad. Sci. U.S.A. 1988; 85:2444–2448. 3162770 10.1073/pnas.85.8.2444 PMC280013

[61] Zhang S. , Krieger J.M., Zhang Y., Kaya C., Kaynak B., Mikulska-Ruminska K., Doruker P., Li H., Bahar I. ProDy 2.0: increased scale and scope after 10 years of protein dynamics modelling with Python. Bioinforma. 2021; 37:3657–3659. 10.1093/bioinformatics/btab187 PMC8545336 33822884

[62] Ritchie D.W. Calculating and scoring high quality multiple flexible protein structure alignments. Bioinforma. 2016; 32:2650–2658. 10.1093/bioinformatics/btw300 27187202

[63] Zhang C. , Zhang X., Freddolino P.L., Zhang Y. BioLiP2: an updated structure database for biologically relevant ligand-protein interactions. NucleicAcids Res. 2023; 10.1093/nar/gkad630. PMC10767969 37522378

