# Supplementary material for: ATLAS: protein flexibility description from atomistic molecular dynamics simulations
Source: Nucleic Acids Res. 2023 Nov 20;52(D1):D384–92. doi: 10.1093/nar/gkad1084 (PMC10767941; doi:10.1093/nar/gkad1084)
Supplement: gkad1084_Supplemental_File [file gkad1084_supplemental_file.pdf]

ATLAS: protein flexibility description from atomistic molecular dynamics simulations

Supplementary Data

Yann Vander Meersche[1], Gabriel Cretin[1], Aria Gheeraert[1], Jean-Christophe Gelly[1]*, and Tatiana Galochkina[1]*

[1] Université Paris Cité and Université des Antilles and Université de la Réunion, INSERM, BIGR, F-75014 Paris, France

* To whom correspondence should be addressed. Tel: +33 1 81 72 43 30; Email: tatiana.galochkina@u-paris.fr.

Correspondence may also be addressed to Jean-Christophe Gelly. Tel: +33 1 81 72 43 23; Email: jean-christophe.gelly@u-paris.fr.

Present Address: BIGR, 8 rue Maria Helena Vieira Da silva, Site Ady Steg, 75014 Paris, France

Website and API implementation **1**
ATLAS database content **2**
Examples of the protein page analysis **6**
Example 1: Analysis of human HLA class I histocompatibility antigen 6
Example 2: Protein containing a dual personality fragment (DPF) 10

Website and API implementation

The front-end of the database website is statically built using the Bootstrap 5 framework (https://getbootstrap.com), in combination with Javascript/jquery. Efficient database searches are facilitated by DataTables . For presenting NCBI-like sequence alignments from BLAST, we employ BlasterJS [1]. For visualising protein structure and dynamics, we utilise the PDBe implementation of Mol* [2]. Additionally, the Saguaro 1D Feature Viewer, developed by RCSB PDB [3], is integrated into the website as well. Interactive graphs are presented using Plotly JS (https://plotly.com/javascript) and Chart.js (https://www.chartjs.org).

To simplify maintenance, the back-end operates as a Docker image with Python 3, executing the scripts responsible for protein research within the database.

The REST API, enabling programmatic access to all downloadable data available on the database, was implemented in Python 3 using the FastAPI framework (https://fastapi.tiangolo.com) encapsulated into a Docker container for better maintainability.

References:
1. Blanco-Míguez,A., Fdez-Riverola,F., Sánchez,B. and Lourenço,A. (2018) BlasterJS: A novel interactive JavaScript visualisation component for BLAST alignment results. *PloS One*, **13**, e0205286.

2. Sehnal,D., Bittrich,S., Deshpande,M., Svobodová,R., Berka,K., Bazgier,V., Velankar,S., Burley,S.K., Koča,J. and Rose,A.S. (2021) Mol* Viewer: modern web app for 3D visualization and analysis of large biomolecular structures. *Nucleic Acids Res.*, **49**, W431–W437.

3. Segura,J., Rose,Y., Westbrook,J., Burley,S.K. and Duarte,J.M. (2021) RCSB Protein Data Bank 1D tools and services. *Bioinforma. Oxf. Engl.*, **36**, 5526–5527.

ATLAS database content

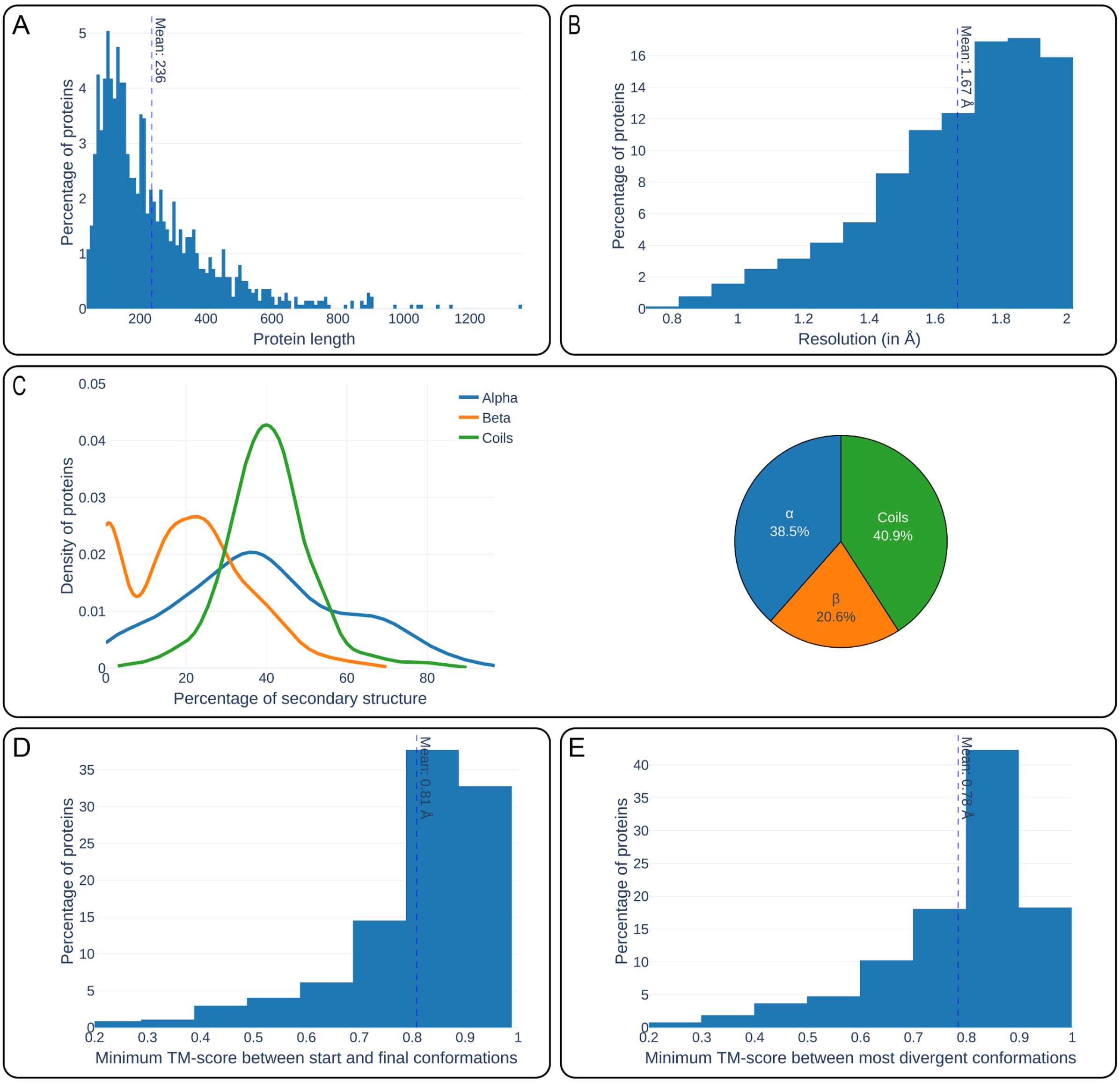

Figure S1: *General structural and dynamical properties in ATLAS main database. Protein length (A) and resolution (B) distributions, secondary structure content (C) and protein structure deviation from the starting conformation of the final (D) and most divergent (E) structures.*

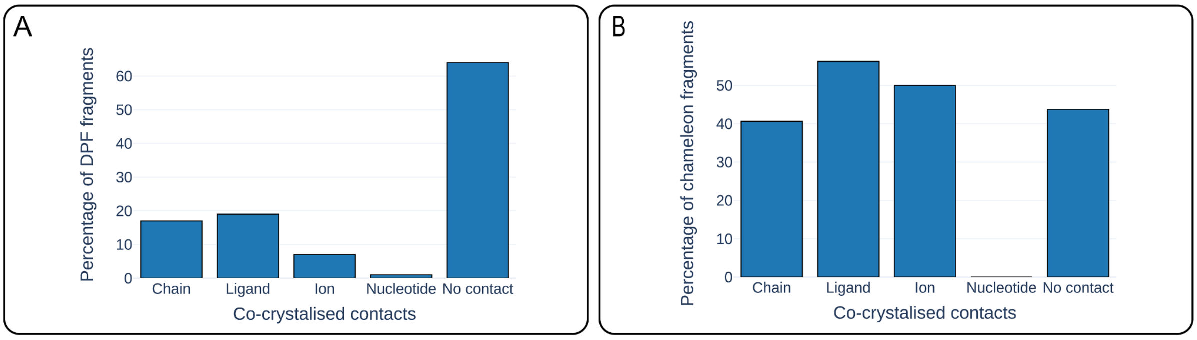

Figure S2: Number of co-crystallised contacts for protein fragments with particular dynamics: *DPF (A) and chameleon fragments (B). Fragments can be involved in multiple co-crystallised contacts.*

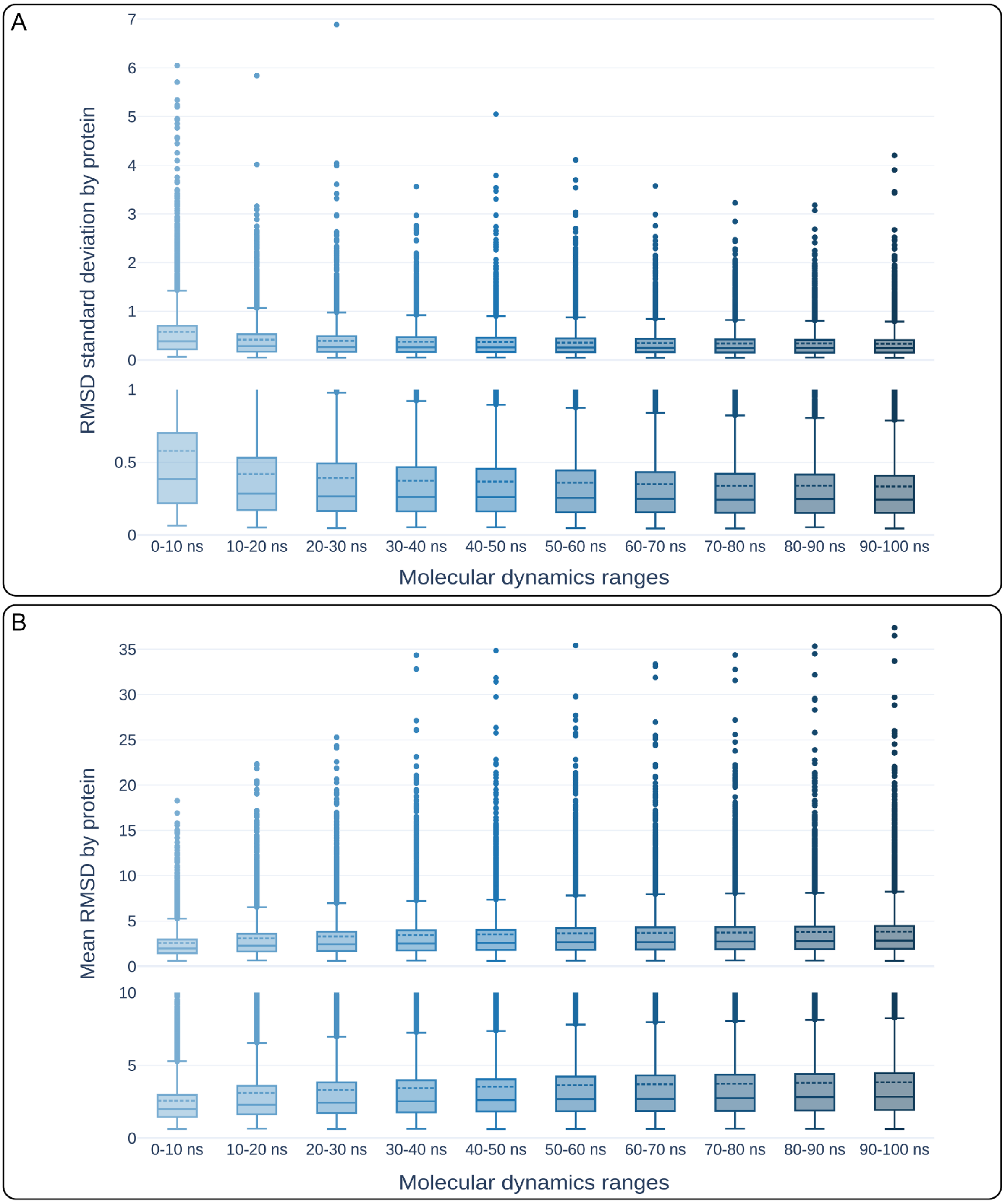

Figure S3: *RMSD evolution through the MD of ATLAS dataset proteins (in Å). Evolution of the RMSD standard deviation (A) and average RMSD (B) during 10 ns ranges. MD simulation replicates were treated separately. Bottom of each plot is zoomed in.*

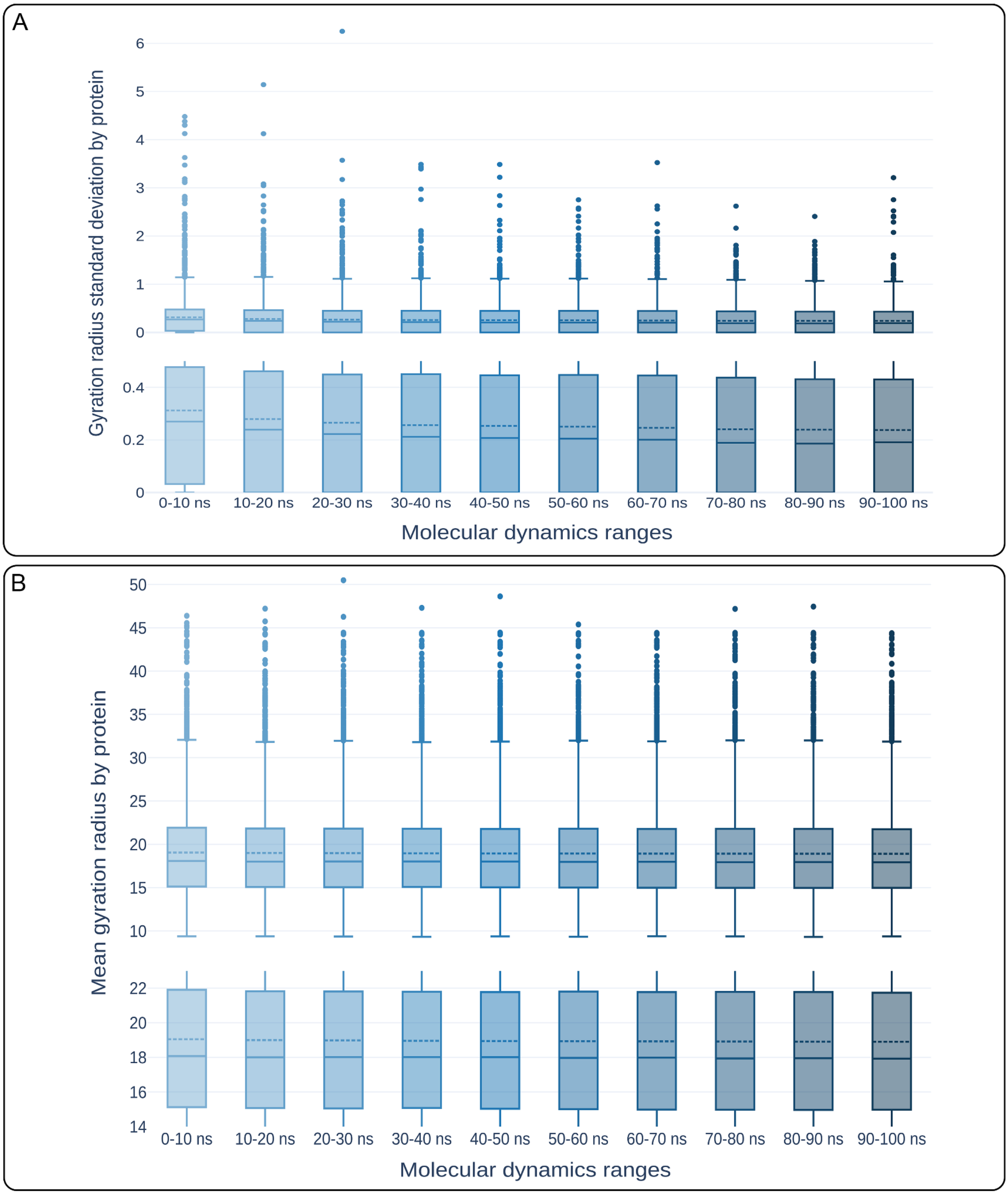

Figure S4: *Gyration radius evolution through the MD of ATLAS dataset proteins (in Å). Evolution of the gyration radius standard deviation (A) and average of gyration radius (B) during 10 ns ranges. MD simulation replicates were treated separately. Bottom of each plot is zoomed in.*

Examples of the protein page analysis

Example 1: Analysis of human HLA class I histocompatibility antigen

https://www.dsimb.inserm.fr/ATLAS/database/ATLAS/1k5n_A/1k5n_A.html

Here we present an example interpretation of the ATLAS entry for a *human HLA class I histocompatibility antigen* (PDB code 1K5N chain A).

The page header informs us that the protein is a Major Histocompatibility Complex (MHC) composed of two ECOD domains (Fig. S5, 1), with an overall content in β-sheets of 39%. We can also see that the minimum TM-score between the conformations is high (greater than 0.5 on the standard scale), indicating that the dynamic deviates from the starting position, but still remains in the same fold (Fig. S5, 2).

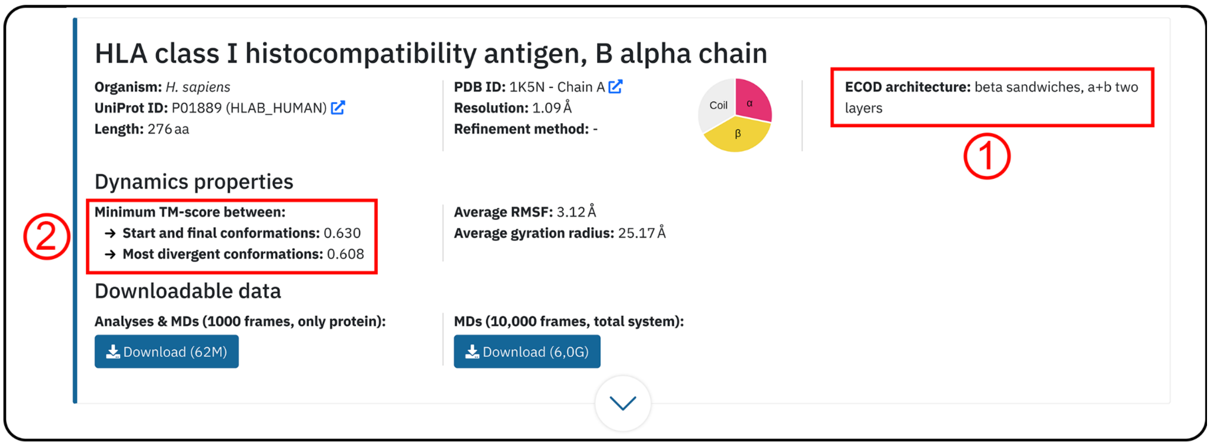

Figure S5: *Page header (PDB code 1K5N chain A).*

Moving on to the general properties section, we find that the protein was co-crystallised in interaction with another chain and a glycerol molecule (Fig. S6, 3). These interactions likely account for the difference in peak intensities between the RMSF and B-factor, especially in the second domain (Fig. S6, 4).

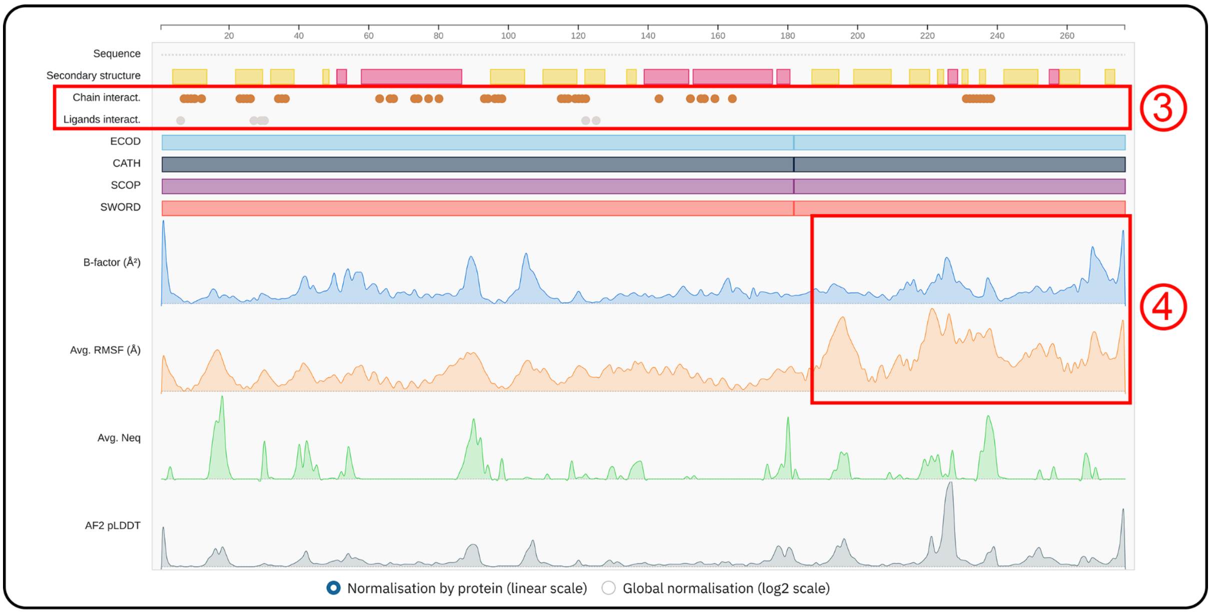

Figure S6: *General properties (PDB code 1K5N chain A).*

The replicates overview reveals a significant conformational change in the first replicate (blue curve), as evidenced by both the RMSD and gyration radius (Fig. S7A, 5-6). This change results from the extension of the protein structure, leading to an increase in the gyration radius in the corresponding trajectory.

Analysing the RMSF plot, we observe pronounced fluctuations in residues 180-276, corresponding to the second beta-sheet domain (Fig. S7B, 7). However, RMSF alone does not provide insights into the local deformability of the backbone. To identify the hinge zones responsible for the conformational variability of the protein structure, we can examine the Protein Blocks Neq values. This analysis highlights the regions around residues 173-184 and 235-240 as undergoing the most pronounced deformations within the region of interest (Fig. S7B, 8), while the region 185-217 appears locally rigid with moderate loop deformability.

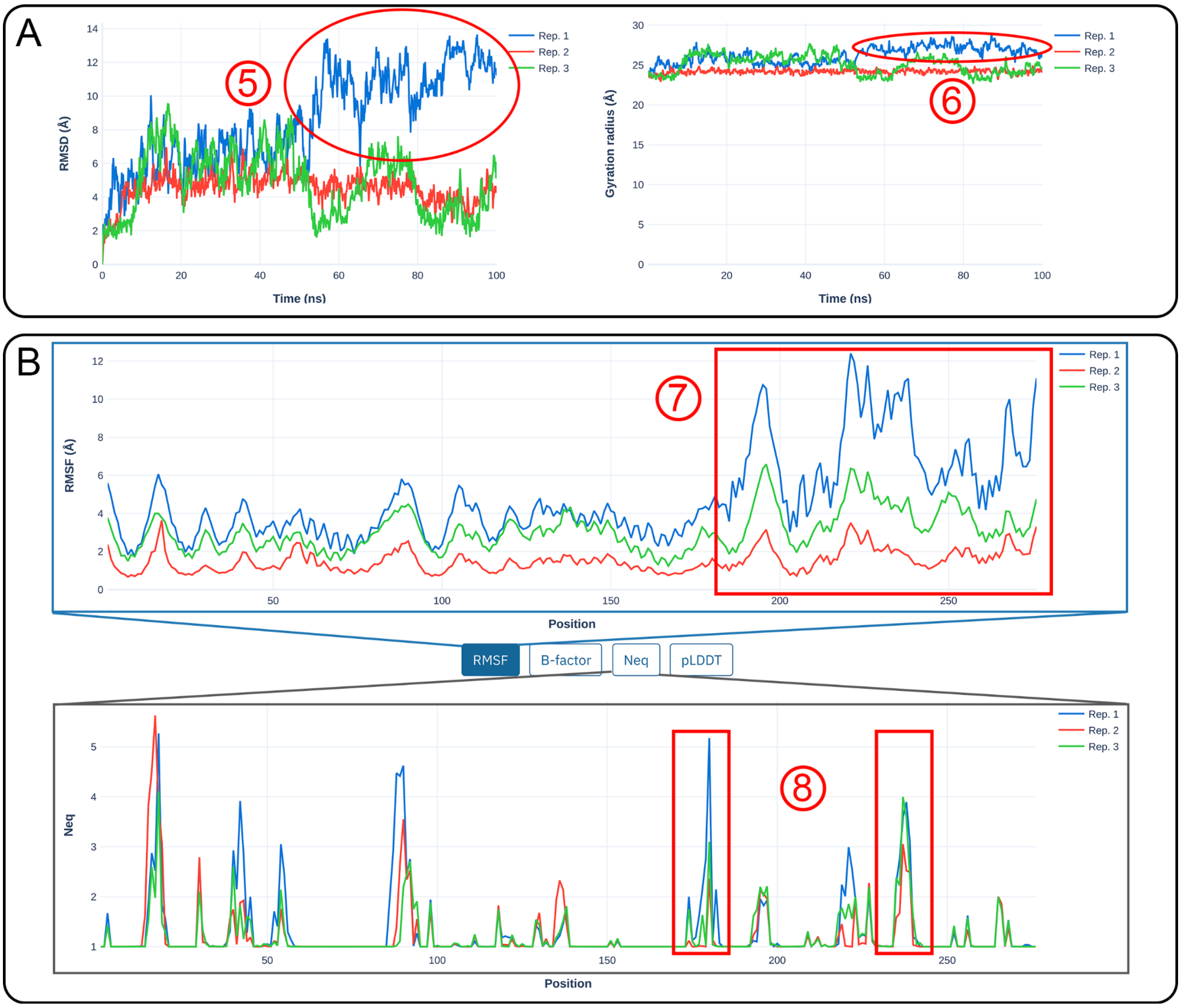

Figure S7: *Replicates overview (PDB code 1K5N chain A).* ***A.*** *RMSD curves (left) and Gyration radius curves (right) for the three replicates.* ***B.*** *RMSF curves (top) and Neq curves (bottom) for the three replicates.*

Finally, the detailed analysis section dives deeper into the analysis of the protein dynamics. We observe that the frequency contact map of the 1st replicate lacks inter-domain contacts (Fig. S8A, 9), unlike replicates 2 and 3, which exhibit a hydrogen bond between ASP30 and ASP238 throughout 97% and 65% of the dynamics, potentially stabilising the two domains. Additionally, the 3D visualisation of the dynamics and DSSP plot reveals that the destabilisation in the first replicate could be attributed to the loss of the alpha-helix at residues 173-184, with a secondary structure alternating between alpha-helix, beta-turn, 3-10 helix, and bend form (Fig. S8B-C, 10), along with the presence of two prolines at residues 184-185, causing the rotational motion of the second beta-sheet domain that can be seen on the 3D visualisation of the dynamics.

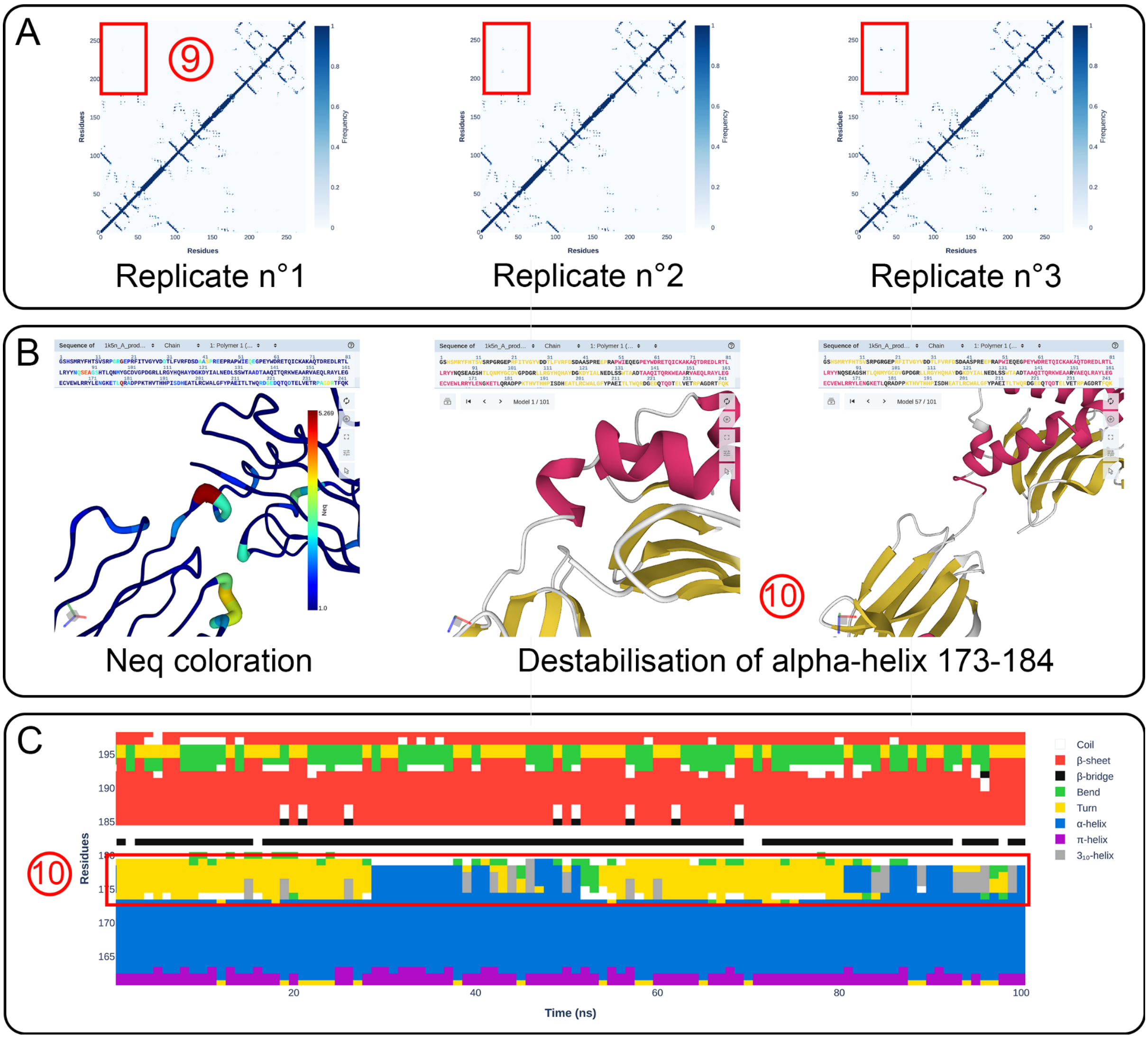

Figure S8: *Detailed analysis (PDB code 1K5N chain A).* ***A.*** *Frequency contact maps of replicates.* ***B.*** *Protein visualisation of replicate n°1.* ***C.*** *DSSP plot of replicate n°1.*

Example 2: Analysis of a hypoxanthine-guanine phosphoribosyltransferase

https://www.dsimb.inserm.fr/ATLAS/database/DPF/1bzy_A/1bzy_A.html

Here we report an example analysis of the *hypoxanthine-guanine phosphoribosyltransferase*, which makes part of the DPF dataset (PDB code 1BZY chain A). HGPRT is an object of intensive biomedical studies due to its role in the development of Lesch–Nyhan syndrome, as well as its abundant presence on the surface of cancer cells making it an important biomarker and potential target for anticancer therapy [1]. In the crystal structure the dual personality fragment is resolved as an ordered antiparallel beta-sheet, covering the enzyme's active site during the enzyme transition-state [2]. Here we summarise information available in the ATLAS database.

According to the page header (Fig. S9), the protein is a human transferase composed of a single a/b three-layered sandwich, with a DPF mainly structured as a beta-sheet. Dual Personality Fragments are regions too flexible to be resolved without a stabilising partner. At the same time, the minimum TM-score between the protein conformations is very high (over 0.8), and the average RMSF is only 1.1 Å therefore indicating modest deviation of the global protein structure during MD simulation.

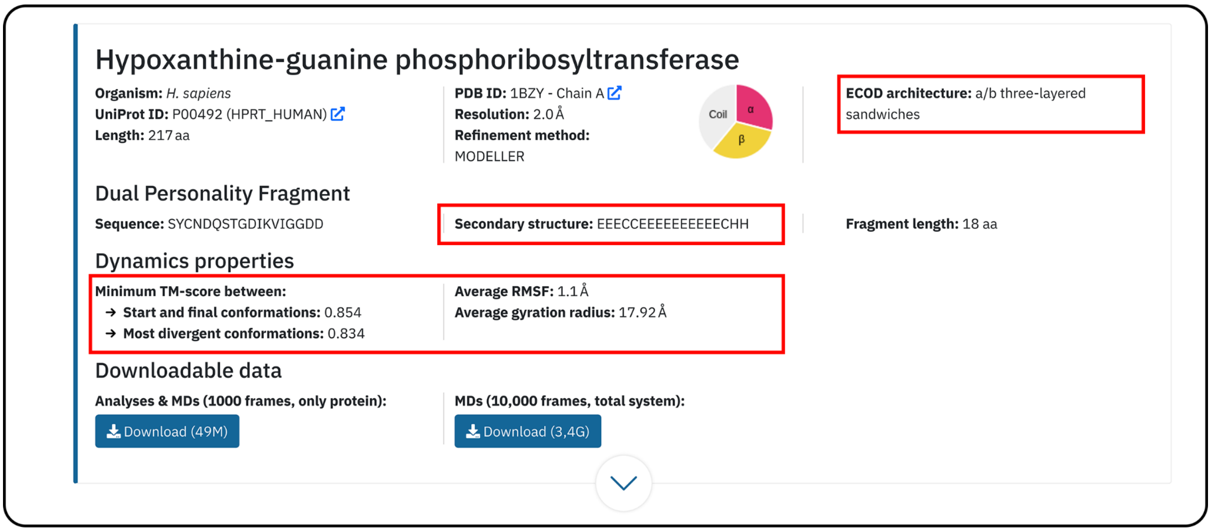

Figure S9: *Page header (PDB code 1BZY chain A).*

Despite the relative stability of the protein general fold, a closer look indicates particular behaviour of the DPF fragment. The general properties section (Fig. S10) confirms that the protein is co-crystallised with other protein chains, ligands and ions, leading to low B-factor values in the DPF region at residues 103-120. However, it appears that B-factors do not

correlate well with the average RMSF and AF2 pLDDT values in this region. While the initial structure of the protein is a beta-sheet, the RMSF obtained in simulations without partners denote a very flexible region and the pLDDT values show an uncertainty in the structure of the predicted region.

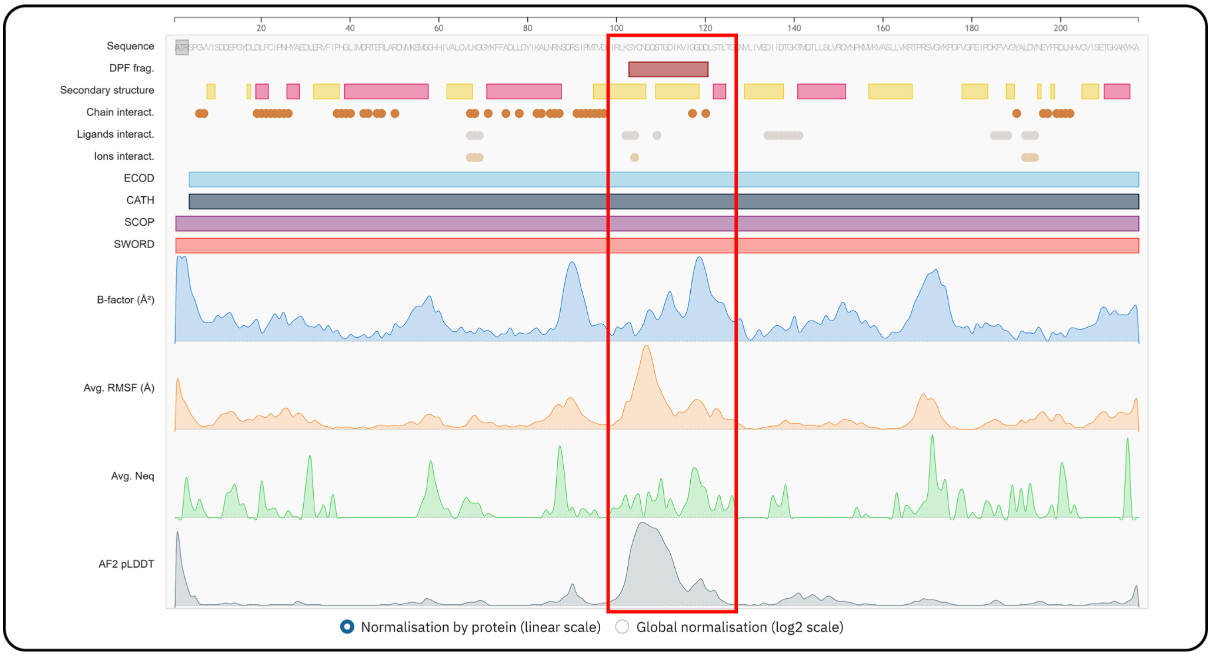

Figure S10: *General properties (PDB code 1BZY chain A).*

The replicate overview section (Fig. S11) confirms these observations, with the first replicate showing the most pronounced flexibility, resulting in an increase in RMSD and gyration radius. Therefore, we will focus on this replicate for further analysis.

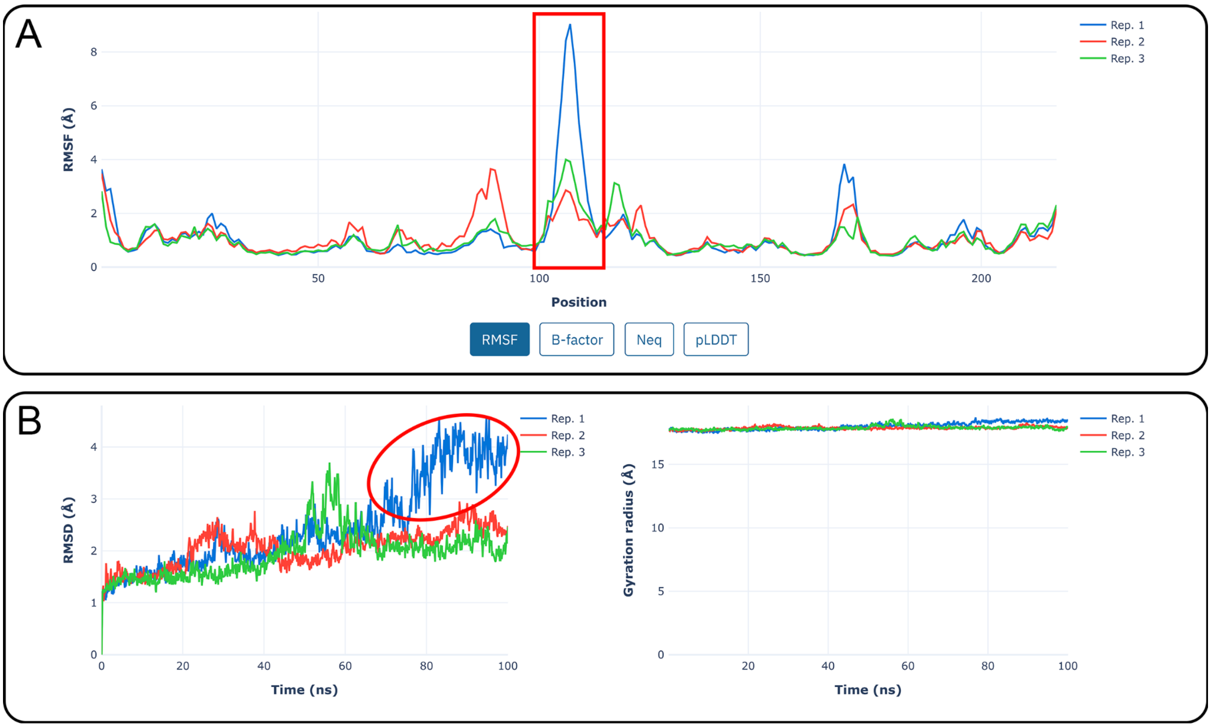

Figure S11: *Replicates overview (PDB code 1BZY chain A).* ***A.*** *RMSF curves for the three replicates.* ***B.*** *RMSD curves (left) and Gyration radius curves (right) for the three replicates.*

By inspecting the protein dynamics in the 3D viewers in the detailed analysis section (Fig. S12), we observe that the DPF can adopt different conformations corresponding to its destabilisation in the absence of ligand partner. The protein's molecular dynamic starts in a compact form and transitions into a more open form explaining the DPF flexibility, correlated with the loss of contacts in the animated contact map, revealing the enzyme's ability to adopt a wide range of conformations in solution. Additionally, the Ramachandran plot confirms that all these motions occur without sampling outlier conformations in terms of dihedral angle values.

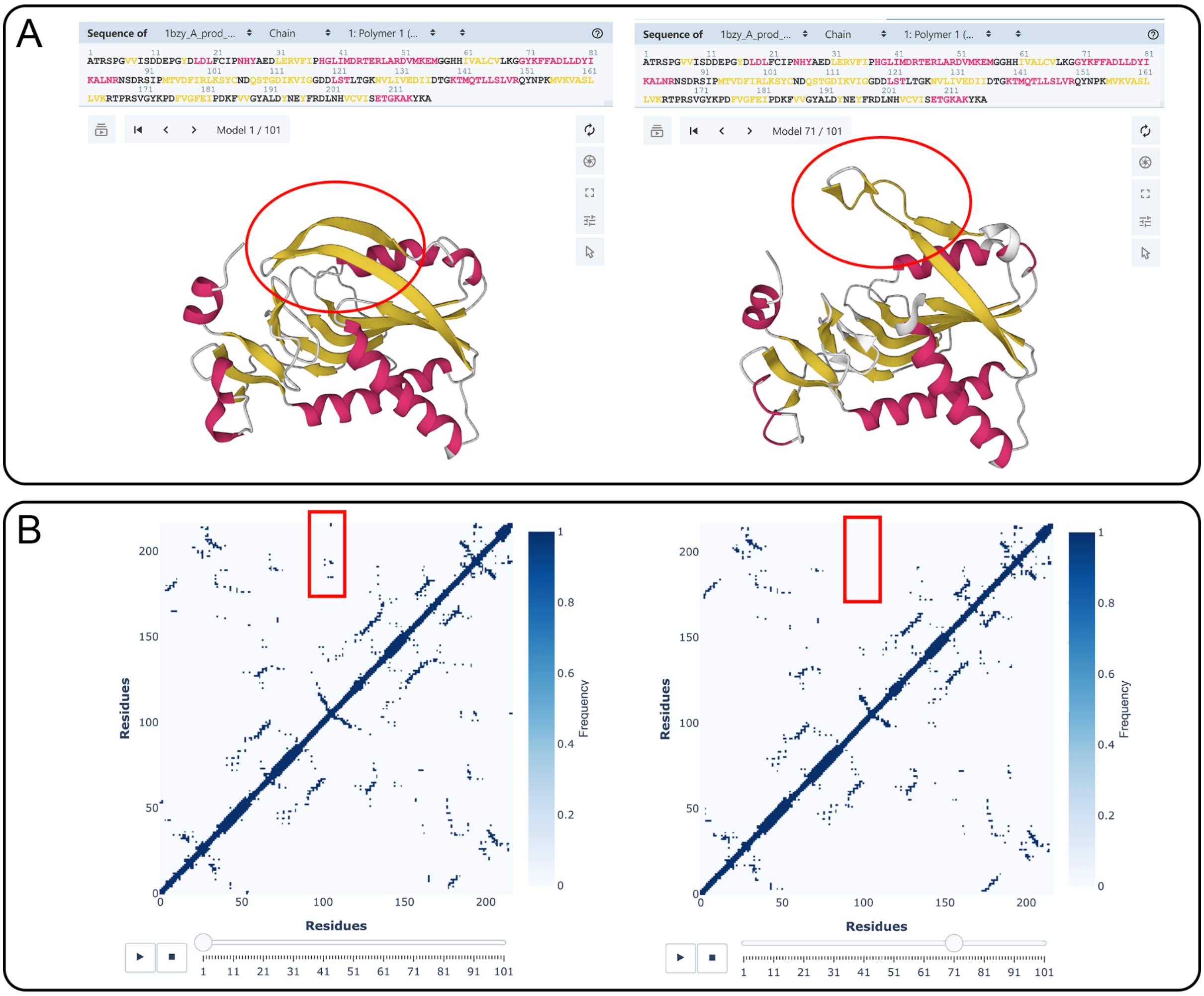

Figure S12: *Detailed analysis (PDB code 1BZY chain A).* ***A.*** *Protein visualisation of the 1st frame of the dynamics (left) and the 71th one (right),* ***B.*** *Animated contact maps at the 1st frame of the dynamics (left) and the 71st one (right).*

Finally, MD simulations and their analysis provided in ATLAS allow us to describe the details of the corresponding conformational transition, which could not be obtained neither in X-ray experiment nor through the analysis of AlphaFold predictions. Obtained conformational ensembles can be further used as starting points for such downstream tasks as drug design using virtual screening for the ensemble of the sampled conformations.

References:

1. Townsend MH, Robison RA, O'Neill KL. A review of HPRT and its emerging role in cancer. Med Oncol. 2018 May 5;35(6):89.

2. Shi W, Li CM, Tyler PC, Furneaux RH, Grubmeyer C, Schramm VL, Almo SC. The 2.0 A structure of human hypoxanthine-guanine phosphoribosyltransferase in complex with a transition-state analog inhibitor. Nat Struct Biol. 1999 Jun;6(6):588-93
